# Magnetic Fano resonances by design in symmetry broken THz meta-foils

**DOI:** 10.1038/srep41869

**Published:** 2017-02-02

**Authors:** Jianfeng Wu, Herbert O. Moser, Rujiang Li, Yihao Yang, Liqiao Jing, Hongsheng Chen, Mark B. H. Breese

**Affiliations:** 1 Department of Physics, National University of Singapore, 2 Science Drive 3, 117542 Singapore; 2 Department of Electrical and Computer Engineering, National University of Singapore, 4 Engineering Drive 3, 117576 Singapore; 3 Karlsruhe Institute of Technology (KIT), Institute of Microstructure Technology (IMT), Postfach 3640, 76021 Karlsruhe, Germany; 4 State Key Laboratory of Modern Optical Instrumentation, Zhejiang University, Hangzhou 310027, China; 5 The Innovative Institute of Electromagnetic Information and Electronic Integration, College of Information Science & Electronic Engineering, Zhejiang University, Hangzhou 310027, China; 6 The Electromagnetics Academy at Zhejiang University, Zhejiang University, Hangzhou 310027, China; 7 Singapore Synchrotron Light Source (SSLS), National University of Singapore, 5 Research Link, 117603 Singapore

## Abstract

Magnetic Fano resonances in there-dimensional symmetry broken meta-foils at THz frequencies are theoretically and experimentally studied. Sharp Fano resonances occur due to the interference between different resonances and can be designed by choosing geometric parameters of the meta-foil. At the Fano resonances, the meta-foil supports antisymmetric modes, whereas, at the main resonance, only a symmetric mode exists. The meta-foil is left-handed at the Fano resonances and shows sharp peaks of the real part of the refractive index in transmission with small effective losses opening a way to very sensitive high-speed sensing of dielectric changes in the surrounding media and of mechanical configuration.

Fano resonance is a fundamental interference phenomenon exhibited by various different oscillating systems encountered in many fields including quantum physics^1^, classical electromagnetism^2^, and acoustics^3^. It is characterized by a peculiar asymmetric profile. Its physical origin is the superposition of a specific narrow band resonance peak on a broad band resonance peak, which leads to constructive interference on the low-frequency side and to destructive interference on the high-frequency side. Over the past years, Fano-type resonances in metamaterials were extensively studied^4^,^5^,^6^,^7^,^8^,^9^,^10^,^11^,^12^. They were exclusively electrically excited in two-dimensional (2D) metamaterials while, for three-dimensional (3D) metamaterials in the THz range, it was considered a challenge to demonstrate Fano resonances^6^. Here, we show that the meta-foil can meet this challenge owing to its fully three-dimensional structure, the magnetic excitation and the highly efficient direct galvanic coupling between resonators. Moreover, the meta-foil offers the inclusion of multiple Fano resonances in one specific meta-foil depending on design.

Meta-foils are three-dimensional double-negative metamaterials transmitting electromagnetic waves at characteristic resonance frequencies. They originate from S-string metamaterials, first introduced in the GHz^13^ domain and later extended to THz frequencies^14^. When such THz S-strings are transversally connected to each other at selected locations^15^,^16^, free-standing all-metal thin-layer space grids are formed. Being locally stiff, yet globally flexible, they can wrap around objects like foils. The meta-foil is unique among flexible metamaterials^17^ as it is all-metal and free-standing. So, the absence of any supporting or embedding dielectric material enables the meta-foil to withstand high temperatures only limited by the melting point of the structural metal, which avoids slowing down of resonance frequencies by dielectric capacitive load, and results in excellent resistance to moisture, chemicals, and ionizing radiation. Meanwhile, a great many of different structures and applications of meta-foils were presented^18^,^19^,^20^,^21^,^22^.

The geometry of meta-foils lends itself not only to varying resonance peaks by design, but also to create a wealth of resonances in one meta-foil by either introducing different cell sizes^19^ or by altering the sequence of interconnecting lines or by combining both. The influence of the sequence of interconnecting lines is the topic of this study as it leads to the occurrence of Fano resonance peaks that can be designed. Fano resonances can be achieved by introducing additional resonators with different resonance frequencies. Such additional resonators can be made by increasing the distance between interconnecting lines which breaks translational symmetry.

In this paper, Fano resonances in 3D symmetry broken meta-foils in the THz range are theoretically and experimentally investigated. Fano resonances in the meta-foil are excited by the magnetic field of the incident wave acting on galvanically coupled resonators. At the Fano resonances, the meta-foil supports antisymmetric modes, whereas, at the main resonance, only a symmetric mode exists. The meta-foil is left-handed at the Fano resonances and shows sharp peaks of the real part of the refractive index in transmission with small effective losses. In the following, upon a description of the geometric structure of symmetry broken meta-foils, resonance frequencies are experimentally measured versus distance of interconnecting lines. The occurrence of Fano resonance peaks is demonstrated and analyzed by means of circuit theory, numerical simulation, and retrieval calculations.

## Results and Discussion

**Figure 1:**
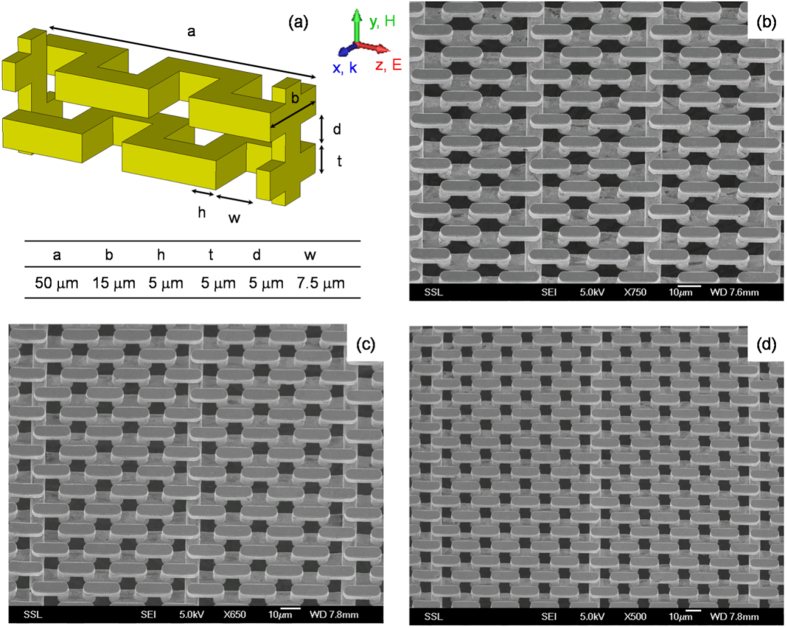
(**a**) Unit cell of a 2SE meta-foil, (**b**–**d**) SEM images of nSE meta-foils with n = 2, 3, 4 made from gold. The electromagnetic wave propagates in x direction.

THz meta-foils were manufactured using three-level lithography with precise alignment and three repeated gold electroplating steps with accurate thickness control^19^,^21^. Sample characterization was carried out by a Bruker IFS 66 v/S Fourier-transform infrared spectrometer^19^,^21^. The radiation source was far infrared synchrotron radiation from ISMI beamline at Singapore Synchrotron Light Source (SSLS)^23^. Transmission spectra were measured by a PE/DLa TGS D201 detector with a spectral resolution of 0.06 THz (2 cm^−1^). In meta-foils, depending on how many S-motifs are included between two subsequent interconnecting lines, structures are called 1SE, 2SE, 3SE, and, in general, *n*SE, where *n* is an integer called order number and E stands for equidistant^16^. Figure 1a and b show a 2SE meta-foil. Experimentally, the focus is on the magnetic resonances of the 2SE meta-foil, although 3SE and 4SE meta-foils are also measured. Their structures are shown in Fig. 1c and d, respectively.

**Figure 2:**
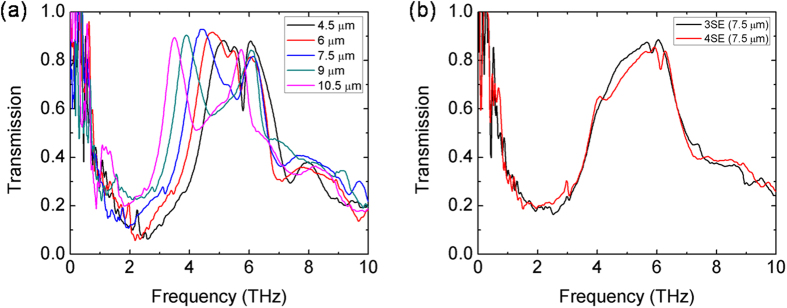
Measured transmission spectra of *n*SE meta-foils for (**a**) *n* = 2 with various loop widths *w*, and (**b**) *n* = 3, 4 with *w* = 7.5 μm.

Figure 2 displays measured transmission spectra of *n*SE meta-foils with *n* = 2, 3, 4 showing the influence of the loop width *w* for the *n* = 2 case (Fig. 2a) and the spectra for *n* = 3, 4 with *w* = 7.5 μm (Fig. 2b). Besides the well-known main magnetic resonance peaks at frequencies *ν*_m_ and the electric resonance peaks, sharp lower frequency magnetic resonance peaks appeared at frequencies *ν*_f_, which are clearly resolved in the 2SE cases, and less so in the 3SE and 4SE cases. According to their profiles, these lower frequency resonances are attributed to Fano resonances as discussed below. The main resonance frequencies *ν*_m_ and Fano resonance frequencies *ν*_f_ of 2SE meta-foils with various loop widths are listed in Table 1.

**Figure 3:**
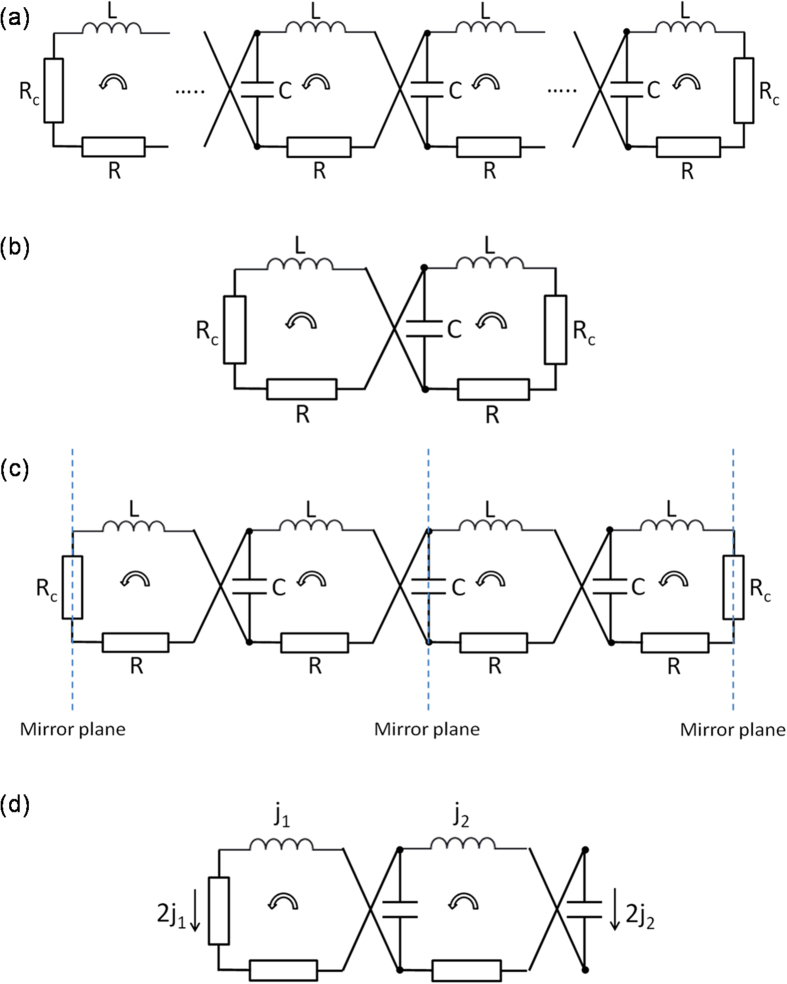
General circuit diagram of (**a**) a *n*SE meta-foil and (**b**) a 1SE meta-foil. (**c**) Unit cell and (**d**) half of unit cell in the circuit diagram of a 2SE meta-foil. The circular arrow indicates the direction of current induced by an increasing magnetic field perpendicular to the drawing plane.

Equivalent circuit theory is used to validate Fano resonances at the lower frequency tails of the transmission spectra. The model circuit diagram of a *n*SE meta-foil is displayed in Fig. 3a showing three types of loops that involve inductance *L*, capacitance *C*, resistance *R* of the S-string proper, and resistance *R*_*c*_ of the interconnecting bar. All cells feature S-string resistance *R* and inductance *L*. In addition, the unit cell comprises a loop including *C* and *R*_*c*_ at the right-hand end, an even number ≥0 of intermediate loops including only *C*, and a termination loop including *R*_*c*_ at the left-hand end.

The 1SE structure is first analyzed as it features only one type of loop formed by a capacitor-resistor cell, as shown in Fig. 3b. The circuit equation reads


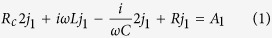


It yields a current *j*_1_ induced by the periodic magnetic flux change *A*_1_ linking the loop that is given by


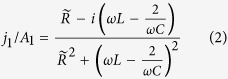


with 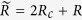. The magnetically excited resonance frequency is


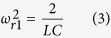


This structure is translationally symmetric for one S length longitudinally. Compared with pure S-strings^13^, the introduction of interconnecting lines does not break the translational symmetry totally, but leaves one S length as a period. As there is only one type of resonant cell in the 1SE structure, there is only one magnetically excited resonance supported by the capacitor-resistor cell.

The circuit scheme of a unit cell of the 2SE meta-foil is displayed in Fig. 3c. It comprises two cells of each type, namely, the two-capacitor and the capacitor-resistor loop. Compared with 1SE, 2SE has an additional two-capacitor cell, so occurrence of two resonances is expected due to the interference between resonances from two different cells. The unit cell of 2SE has two symmetry planes, either through the center capacitor or through the interconnecting resistor. To derive circuit equations, it is sufficient to treat one half of the unit cell, as depicted in Fig. 3d. As the unit cell is symmetric with respect to the interconnecting line and to the central capacitor, the currents flowing through the central capacitor and the interconnecting resistor are 2*j*_2_ and 2*j*_1_, respectively. Hence, the two equations derived from the circuit in Fig. 3d are


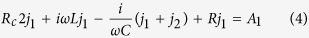



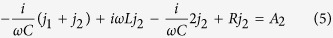


The derivation process is described in detail in Supporting Information. Neglecting resistances yields


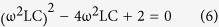


with solutions


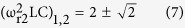


The zero resistance currents are


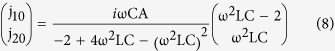


Setting 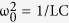 and x = ω/ω_0_, results in


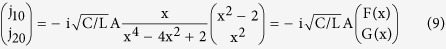


**Figure 4:**
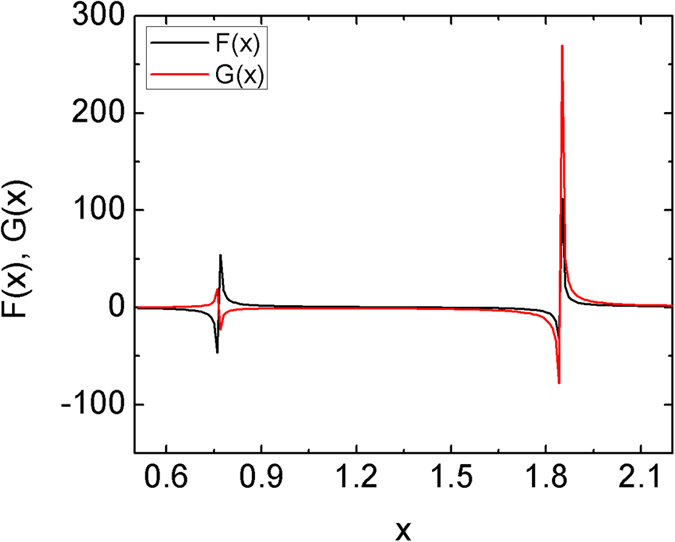
Normalized currents *F(x*) (black) and *G(x*) (red) in the two loops of the half 2SE circuit versus normalized angular frequency *x*. At the lower resonance, currents are in opposite phase whereas they have equal phase at the higher resonance.

Figure 4 shows the spectra of a 2SE meta-foil computed from Equation 9 where F(x) and G(x) are in black and red, respectively. As expected, two different magnetic resonance peaks appear, one at x = 1.87 corresponding to the main magnetic resonance at frequency ν_m_, and the other at x = 0.75 corresponding to the Fano resonance at frequency ν_f_. The Fano resonances result from the interference between resonances from two different cells. As shown in Fig. 1, for the *n*SE meta-foils with *n* > 1, the introduction of interconnecting lines increases the longitudinal period to *n* times the S length, and the translational symmetry no longer holds for one S length. Thus, an additional type of cell which comprises two capacitors is created by the symmetry break in the structure according to the circuit diagram shown in Fig. 3b. The additional resonances induced by the additional type of cells are superposed on the lower frequency tail of the magnetic resonance peak induced by the capacitor-capacitor cell, and lead to the Fano resonances. Moreover, the frequency ratio of the two peaks is given by 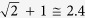 in Equation 7, which is independent of the cell size. The absolute distance between peaks can only be influenced by *ω*_0_, i.e., by capacitance *C* and inductance *L* in the structure. Besides, Fig. 4 reveals the currents having equal phase at the higher main resonance, and opposite phase at the lower Fano resonance. This implies that the 2SE meta-foil may support the symmetric mode at the main resonance, and the antisymmetric mode at the Fano resonance.

The circuit analysis for 3SE and 4SE meta-foils is presented (Supporting Information). In summary, Table 2 shows resonance frequencies for *n*SE meta-foils with *n* = 1, 2, 3, 4, and a comparison with the resonance frequency for the pure S-strings. Interestingly, the main resonance frequency for *n*SE meta-foils tends to the resonance frequency of the pure S-strings, as *n* approaches infinity. Physically, the *n*SE meta-foil with infinite *n* reduces to S-strings, where the interconnection lines at the two boundaries have little impact on the transmission spectrum of the whole structure.

Finally, the general case nSE leads to


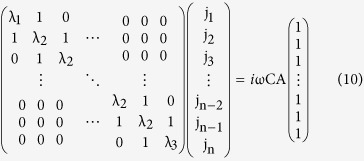


Cramer’s rule^24^ yields the solution


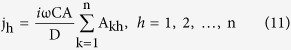


with *D* the determinant of the above matrix and *A*_*kh*_ the algebraic complements to the right-hand side unit vector. The resonances occur at the zeros of *D* which can be found from the secular equation of *D* (Supporting Information).

From the above equivalent circuit analysis, a symmetry broken *n*SE meta-foil exhibits one main resonance and *n*-1 Fano resonances. At the main resonance, the meta-foil supports the symmetric mode while at the Fano resonances, it supports the antisymmetric modes. If *n* approaches infinity, the main resonance frequency of *n*SE meta-foils tends to the resonance frequency of the S-strings. Besides, from a mechanical point of view, when *n* increases, the mechanical strength of the meta-foil decreases, it becomes more flexible. So, symmetry broken *n*SE meta-foils with *n* > 1 are of interest with regard to both supporting multiple Fano resonances and increasing mechanical flexibility.

**Figure 5:**
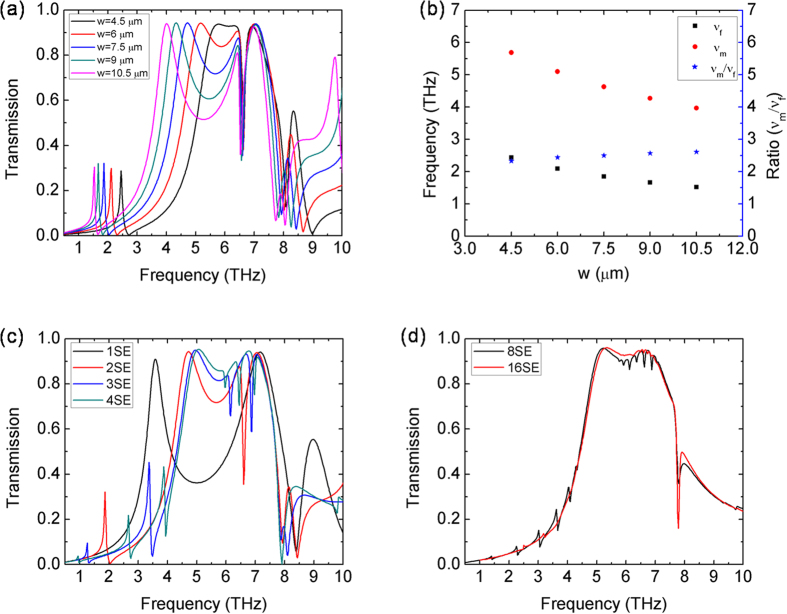
(**a**) Simulated transmission spectra of the 2SE meta-foil with various loop widths. Fano resonance peaks range from about 1.5 to 2.5 THz, main ones from about 4.0 to 5.7 THz, and electric peaks stay roughly at the same frequency of 7 THz. (**b**) Detailed peak frequencies and ratio of main to Fano peaks. Simulated transmission spectra of *n*SE meta-foils with *w* = 7.5 μm and (**c**) *n* = 1, 2, 3, 4, and (**d**) *n* = 8, 16.

Resonance frequencies and modes in *n*SE meta-foils are also numerically analyzed by using the frequency domain solver of the CST Microwave Studio, which implemented a finite element method to determine reflection and transmission properties. In the simulations, the unit cell boundary condition was applied, and the gold was modeled as a lossy metal with conductivity σ = 4.09 × 10^7^ Sm^−1^. Figure 5a displays a series of transmission spectra of 2SE structures with various loop width *w*. There are two magnetic resonance peaks (main and Fano) that vary as a function of *w*, whereas the electric resonance peak stays roughly at the same frequency of 7 THz. The ratio between the two resonance frequencies ranges from about 2.61 to 2.33 for various loop widths (Fig. 5b). Note that these ratios are closer to the experimental ones than the frequencies themselves as given in Table 1. The deviation from the zero-resistance ratio 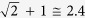 in circuit theory is believed to be due to the coupling between adjacent string pairs accounted for in numerical simulation, thus pulling down frequencies. Therefore, in the 2SE case, the frequency ratio of the magnetic peaks does not depend on the values of L and C, which can be understood by noting that, in different cells, the inductances are identical and the capacitances multiples of one common capacitance. Note that the separation between Fano peaks can be made smaller (or larger) by varying the capacitors. For instance, if the central capacitor is made larger by designing a smaller gap there, then the upper Fano resonance is lowered. Or, if the capacitor in the interconnecting line loop is made smaller, the lower Fano resonance frequency is shifted upwards. So, the main parameters available to design Fano resonances in meta-foils are the number of cells between interconnecting lines n, the width of a loop w, and the capacitor gap.

Figure 5c shows transmission spectra for various structures from 1SE to 4SE where the resonance frequencies are approximately equal to the experimental results. Obviously, the 1SE meta-foil does not exhibit any Fano resonance. The 2SE structure features one Fano resonance peak at 1.86 THz, the 3SE two Fano resonance peaks at about 1.26 THz and 3.37 THz, and, finally, the 4SE three Fano resonance peaks at 0.95 THz, 2.67 THz, and 3.88 THz. The number of main magnetic resonance peaks is always one. So, in this range of n, nSE structures have n-1 Fano peaks and one main resonance peak, in agreement with circuit theory. Meanwhile, for higher n, the amplitudes of the n-1 Fano peaks decrease with n, and the remaining one main resonance tends to the resonance of the pure S-strings, as shown in Fig. 5d. This numerical result agrees with circuit theory as well.

**Figure 6:**
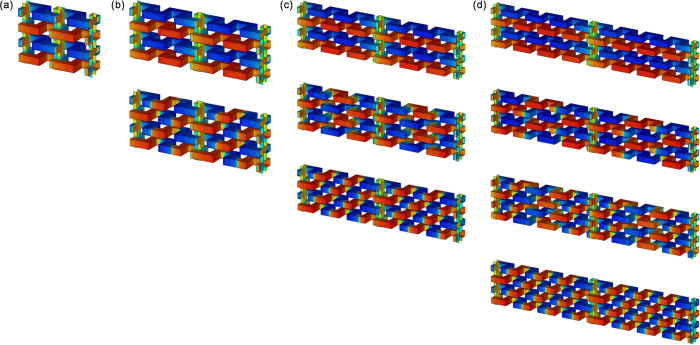
Electric field distributions of 1SE to 4SE (from (**a**) to (**d**)). The parameters are 3.57 THz for 1SE, 1.86 THz and 4.72 THz for 2SE, 1.26 THz, 3.37 THz and 4.95 THz for 3SE, 0.95 THz, 2.67 THz, 3.88 THz and 5.06 THz for 4SE (frequencies from top to bottom).

As discussed in circuit theory, Fano resonances and main resonance in the transmission spectrum correspond to two different modes. At the Fano resonances, currents in different loops have opposite signs (antisymmetric modes), whereas they have equal signs (symmetric mode) at the main resonance frequency. Figure 6 displays a time-domain simulation of the spatial distribution of the electric field in the four cases 1SE to 4SE. Obviously, at the main resonance, the electric field is symmetric in the entire loop between two subsequent interconnection lines, exhibiting the symmetric mode, whereas, with decreasing frequency, the loops’ electric field is antisymmetric with mutually opposite phases, exhibiting the antisymmetric mode. The numerical results validate those obtained from circuit theory.

**Figure 7:**
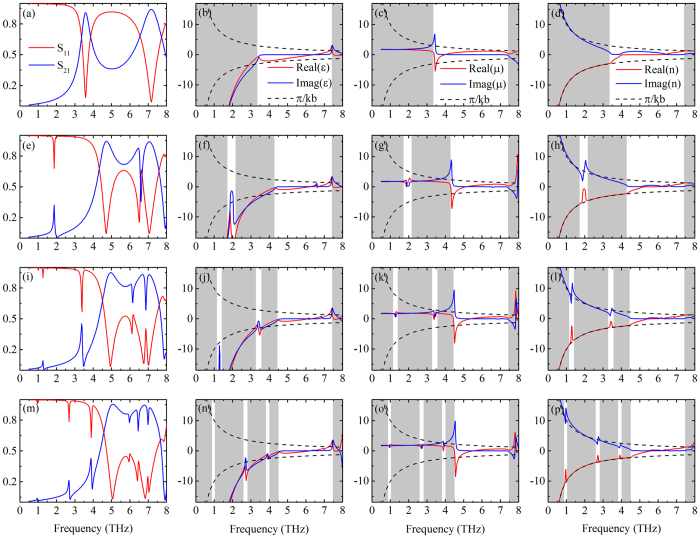
Simulated spectra (first column), retrieval constitutive parameters (second and third columns) and refractive index (last column) for *n* = 1, 2, 3, 4 from top to bottom rows.

While the single resonance peak of the 1SE structure at 3.5 THz was found to be left-handed in earlier retrieval calculations^15^,^19^, the nature of various peaks for higher nSE structures is studied here when n ≥ 2 in the THz frequency range. A key question is whether meta-foils at Fano resonances are also left-handed, as they would fulfill the requirement of an effective medium better since their ratio of wavelength to structure dimension is larger. For instance, the Fano peak of the 2SE meta-foil leads to a wavelength-structure ratio as large as ~10, in the 4SE case even 20. Figure 7 gives an overview of retrieval results from the magnitude and phase of the linear S parameters (S_11_ and S_21_), where shows the S parameters, the retrieved constitutive parameters, and refractive index, respectively. For frequencies smaller than the main magnetic resonance frequency, meta-foils are left-handed because of the negative real part of the refractive index, and the width of the negative index ranges increases with order number n. In the last column of Fig. 7, shaded regions describe where Real(n) reaches the boundary of the Brillouin zone π/kb, and the wavelength inside the metamaterial at the boundary is comparable or smaller than the structure period ruling out the use of the effective medium theory to characterize the meta-foils. As shown in the first column of Fig. 7, both S_11_ and S_21_ exhibit Fano resonances, where S_21_ shows an asymmetric peak-and-dip profile, S_11_ shows a reverse dip-and-peak profile, and the peaks of S_21_ and dips of S_11_ are quite pronounced compared with their dips and peaks, respectively. These two different profiles lead to two different profiles of the real and imaginary parts of the refractive index, as shown in the last column of Fig. 7. The resulting Real(n) has pronounced upward peaks with Imag(n) in their local minima. Especially, at these Fano resonances, Real(n) jumps from the edge of the Brillouin zone right into its interior over its small bandwidth. This intriguing phenomenon is important for the application of left-handed meta-foils. Moreover, these shaper Fano resonances can support many potential applications on sensing and counterfeiting^6^,^15^,^25^,^26^ discussed in Supporting Information.

## Conclusion

In this work, we have theoretically and experimentally studied the magnetic Fano resonances in 3D symmetry broken meta-foils in the THz range. At the Fano resonances, the meta-foil supports antisymmetric modes, whereas, at the main resonance, only a symmetric mode exists. An nSE meta-foil is able to induce one main resonance and n-1 Fano resonances. The ratio between different resonance frequencies is independent on the geometry parameters and might be useful for spectral scaling/calibration in THz spectroscopy. Moreover, the influence of the order number, the loop width, and the capacitor gap opens a way of engineering the Fano resonances in double-negative meta-foils. The meta-foil is left-handed at the Fano resonances and shows sharp peaks of the real part of the refractive index in transmission with small effective losses opening a way to very sensitive high-speed sensing of dielectric changes in the surrounding media and of mechanical configuration.

## Additional Information

**How to cite this article:** Wu, J. *et al*. Magnetic Fano resonances by design in symmetry broken THz meta-foils. *Sci. Rep.* **7**, 41869; doi: 10.1038/srep41869 (2017).

**Publisher's note:** Springer Nature remains neutral with regard to jurisdictional claims in published maps and institutional affiliations.

## Supplementary Material

Supplementary Information

## Figures and Tables

**Table 1:** Main and Fano magnetic resonance frequencies, ν_m_ and ν_f_, respectively, and their ratio versus loop width for 2SE meta-foils.

*w* (μm)	4.5	6	7.5	9	10.5
*ν*_m_ (THz)	5.1	4.7	4.3	3.9	3.4
*ν*_f_ (THz)	2.2	1.9	1.7	1.4	1.2
ν_m_/ν_f_	2.3	2.5	2.5	2.8	2.8

**Table 2:** Resonance frequencies for S-strings and nSE meta-foils for n = 1, 2, 3, 4.

	S-strings	1SE	2SE	3SE	4SE
Secular equation			1−*λ*_13_ = 0	*λ*_123_−*λ*_1_−*λ*_3_ = 0	(*λ*_23_−1)(*λ*_12_−1)−*λ*_13_ = 0
*ω*^2^*LC*	4	2	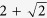	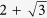	2 + 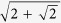
			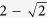	2	2 + 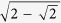
				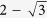	2 − 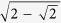
					2 − 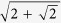
