# Supplementary material for: Magnetic Fano resonances by design in symmetry broken THz meta-foils
Source: Sci Rep. 2017 Feb 2;7:41869. doi: 10.1038/srep41869 (PMC5288773; doi:10.1038/srep41869)
Supplement: Supplementary Information [file srep41869-s1.pdf]

Supporting Information

Magnetic Fano resonances by design in symmetry broken THz meta-foils

Jianfeng Wu[1,2,*], Herbert O. Moser[3], Rujiang Li[4,5,6], Yihao Yang[4,5,6], Liqiao Jing[4,5,6], Hongsheng Chen[4,5,6], and Mark B. H. Breese[7]

Equivalent circuit analysis

For the 2SE meta-foils, the two equations derived from the circuit in Figure 3c are

$$R_c 2j_1 + i\omega L j_1 - \frac{i}{\omega C}(j_1 + j_2) + R j_1 = A_1$$

$$-\frac{i}{\omega C}(j_1 + j_2) + \ i\omega L j_2 - \frac{i}{\omega C} 2j_2 + R j_2 = A_2 \qquad \text{(S1)}$$

In matrix notation, after multiplication by $i\omega C$ and setting $A_i = A$ with i = 1, 2, the circuit equations read

$$\begin{pmatrix} \lambda_1 & 1 \\ 1 & \lambda_3 \end{pmatrix} \begin{pmatrix} j_1 \\ j_2 \end{pmatrix} = i\omega C A \begin{pmatrix} 1 \\ 1 \end{pmatrix} \qquad \text{(S2)}$$

where

$$\lambda_1 = 1 - \omega^2 LC + i\omega C\tilde{R} \qquad \text{(S3)}$$

$$\lambda_3 = 3 - \omega^2 LC + i\omega CR \qquad \text{(S4)}$$

Moreover, setting

$$\lambda = 1 - \omega^2 LC + i\omega CR \qquad \text{(S5)}$$

results in

$$\lambda_1 = \lambda + i\omega C 2R_c \qquad \text{(S6)}$$

$$\lambda_3 = \lambda + 2 \qquad \text{(S7)}$$

Gauss' elimination method leads to

$$\begin{pmatrix} j_1 \\ j_2 \end{pmatrix} = \frac{i\omega CA}{1-\lambda_1\lambda_3}\begin{pmatrix} 1-\lambda_3 \\ 1-\lambda_1 \end{pmatrix} \tag{S8}$$

with resonances obtained from

$$\lambda_1\lambda_3 = 1 = \left(1-\omega^2 LC + i\omega C\tilde{R}\right)(3-\omega^2 LC + i\omega CR) \tag{S9}$$

Neglecting resistances yields

$$(\omega^2 LC)^2 - 4\omega^2 LC + 2 = 0 \tag{S10}$$

with solutions

$$(\omega_{r2}^2 LC)_{1,2} = 2 \pm \sqrt{2}. \tag{S11}$$

The zero resistance currents are

$$\begin{pmatrix} j_{10} \\ j_{20} \end{pmatrix} = \frac{i\omega CA}{-2+4\omega^2 LC-(\omega^2 LC)^2}\begin{pmatrix} \omega^2 LC - 2 \\ \omega^2 LC \end{pmatrix} \tag{S12}$$

Setting $\omega_0^2 = 1/LC$ and $x = \omega/\omega_0$, results in

$$\begin{pmatrix} j_{10} \\ j_{20} \end{pmatrix} = -i\sqrt{C/L}A\frac{x}{x^4-4x^2+2}\begin{pmatrix} x^2-2 \\ x^2 \end{pmatrix} = -i\sqrt{C/L}A\begin{pmatrix} F(x) \\ G(x) \end{pmatrix}. \tag{S13}$$

For higher meta-foils with $n \geq 3$, the number of two-capacitor loops exceeds the number of capacitor-resistor loops increasingly. An nSE cell has $2(n-1)$ two-capacitor loops whereas the number of capacitor-resistor loops stays two, so the ratio of numbers of two-capacitor to capacitor-resistor loops is n-1. These two-capacitor loops will create additional magnetic resonances, which are responsible for the occurrence of Fano resonances.

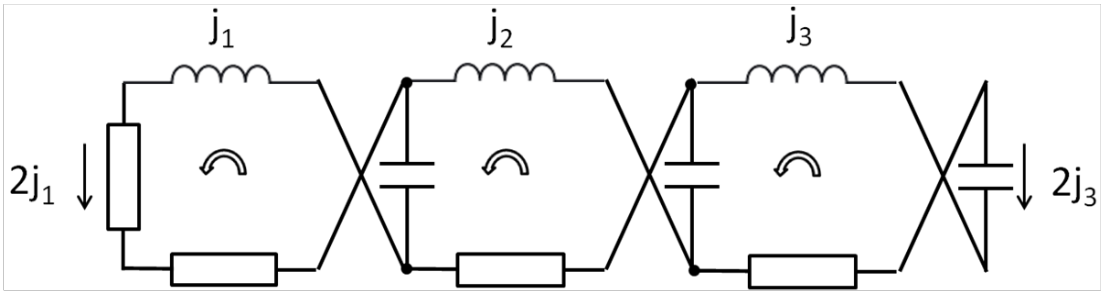

Figure S1. Half unit cell of the 3SE meta-foil.

According to Figure S1, the circuit equations for 3SE are

$$R_c 2j_1 + i\omega L j_1 - \frac{i}{\omega C}(j_1 + j_2) + R j_1 = A_1$$

$$-\frac{i}{\omega C}(j_1 + j_2) + \ i\omega L j_2 - \frac{i}{\omega C}(j_2 + j_3) + R j_2 = A_2$$

$$-\frac{i}{\omega C}(j_2 + j_3) + \ i\omega L j_3 - \frac{i}{\omega C} 2j_3 + R j_3 = A_3 \qquad \text{(S14)}$$

and the matrix form is

$$\begin{pmatrix} \lambda_1 & 1 & 0 \\ 1 & \lambda_2 & 1 \\ 0 & 1 & \lambda_3 \end{pmatrix} \begin{pmatrix} j_1 \\ j_2 \\ j_3 \end{pmatrix} = i\omega C A \begin{pmatrix} 1 \\ 1 \\ 1 \end{pmatrix} \qquad \text{(S15)}$$

where $\lambda_2 = \lambda_3 - 1$, indicating that there are only two different types of loops. Solving again by Gauss' elimination method, we obtain

$$\begin{pmatrix} j_1 \\ j_2 \\ j_3 \end{pmatrix} = \frac{i\omega C A}{\lambda_1 \lambda_2 \lambda_3 - \lambda_1 - \lambda_3} \begin{pmatrix} \lambda_3(\lambda_2 - 1) \\ \lambda_1(\lambda_3 - 1) - \lambda_3 \\ \lambda_1(\lambda_2 - 1) \end{pmatrix} \qquad \text{(S16)}$$

Resonances occur at $\lambda_1 \lambda_2 \lambda_3 - \lambda_1 - \lambda_3 = 0$ and there are three solutions as expected. Neglecting resistances as above, we get

$$(1 - x^2)(2 - x^2)(3 - x^2) - 1 + x^2 - 3 + x^2 = 0$$

$$(2 - x^2)(x^4 - 4x^2 + 1) = 0 \qquad \text{(S17)}$$

with the solutions $x_1^2 = 2$ and $x_{2/3}^2 = 2 \pm \sqrt{3}$. The zero-resistance currents are

$$\begin{pmatrix} j_{10} \\ j_{20} \\ j_{30} \end{pmatrix} = \frac{i\omega CA}{\lambda_1\lambda_2\lambda_3-\lambda_1-\lambda_3} \begin{pmatrix} \lambda_3(\lambda_2-1) \\ \lambda_1(\lambda_3-1)-\lambda_3 \\ \lambda_1(\lambda_2-1) \end{pmatrix} =$$

$$i\sqrt{C/L}A\,\frac{x}{(2-x^2)(x^4-4x^2+1)} \begin{pmatrix} (3-x^2)(1-x^2) \\ (1-x^2)(2-x^2)-3+x^2 \\ (1-x^2)^2 \end{pmatrix} = i\sqrt{\frac{C}{L}}A \begin{pmatrix} F(x) \\ G(x) \\ H(x) \end{pmatrix} \qquad \text{(S18)}$$

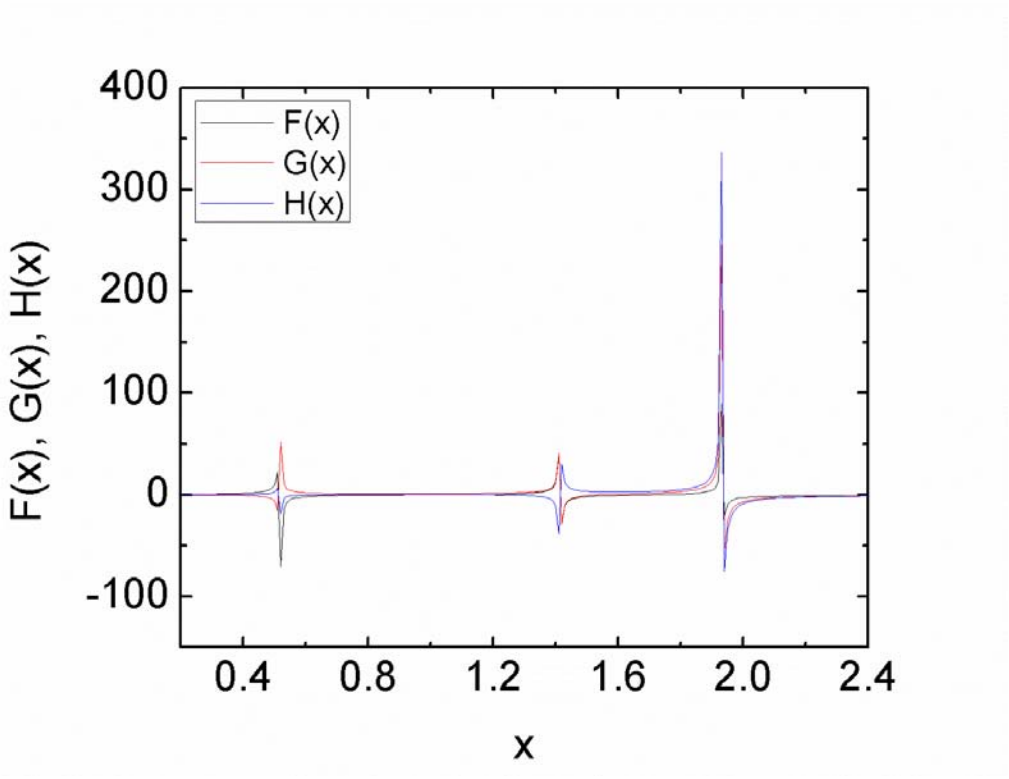

Figure S2. Normalized currents F(x) (black), G(x) (red), and H(x) (blue) in the three loops of the half 3SE circuit versus normalized angular frequency x.

Figure S2 shows the spectrum of such a 3SE meta-foil as calculated from Equation S18, where $F(x)$, $G(x)$ and $H(x)$ are in black, red, and blue, respectively. As expected, three different magnetic resonance peaks are shown, where the resonance peak at x = 1.9 corresponds to the main magnetic resonance peak in Figure 2b, and the resonance peaks at x = 1.4 and x = 0.5 correspond to the Fano resonance peaks. Similarly, the Fano resonances in the symmetry broken 3SE meta-foil are the results of the superposition

between the additional resonances and the magnetic resonance induced by the capacitor-resistor cell.

Moreover, the frequency ratios of the three peaks are also fixed, which are independent on the size of the cell. Besides, as shown in Figure S2, the three currents have equal phases at the highest main resonance frequency, whereas only two of the three currents have equal phases at the lower Fano resonance frequencies. This implies that the 3SE meta-foil may only support the symmetric mode at the main resonance frequency, and the modes are both antisymmetric at the Fano resonance frequencies.

Fourthly, the circuit equations for 4SE are

$$R_c 2j_1 + i\omega L j_1 - \frac{i}{\omega C}(j_1 + j_2) + Rj_1 = A_1$$

$$-\frac{i}{\omega C}(j_1 + j_2) + i\omega L j_2 - \frac{i}{\omega C}(j_2 + j_3) + Rj_2 = A_2$$

$$-\frac{i}{\omega C}(j_2 + j_3) + i\omega L j_3 - \frac{i}{\omega C}(j_3 + j_4) + Rj_3 = A_3$$

$$-\frac{i}{\omega C}(j_3 + j_4) + i\omega L j_4 - \frac{i}{\omega C}2j_4 + Rj_4 = A_4 \qquad \text{(S19)}$$

and the matrix form is

$$\begin{pmatrix} \lambda_1 & 1 & 0 & 0 \\ 1 & \lambda_2 & 1 & 0 \\ 0 & 1 & \lambda_2 & 1 \\ 0 & 0 & 1 & \lambda_3 \end{pmatrix} \begin{pmatrix} j_1 \\ j_2 \\ j_3 \\ j_4 \end{pmatrix} = i\omega CA \begin{pmatrix} 1 \\ 1 \\ 1 \\ 1 \end{pmatrix} \qquad \text{(S20)}$$

We can see that when we increase *n*, we do it by intercalating more inner lines in the system of linear equations. We find

$$\left(\lambda_1 - \frac{1}{\lambda_2 - \frac{1}{\lambda_2 - \frac{1}{\lambda_3}}}\right)\left(\lambda_2 - \frac{1}{\lambda_2 - \frac{1}{\lambda_3}}\right)\left(\lambda_2 - \frac{1}{\lambda_3}\right)\lambda_3 = \lambda_{1223} - \lambda_{12} - \lambda_{13} - \lambda_{23} + 1 = 0 \qquad \text{(S21)}$$

using the notation $\lambda_i\lambda_j\lambda_k\lambda_l = \lambda_{ijkl}$. Resonances occur at $(\lambda_{23}-1)(\lambda_{12}-1)-\lambda_{13}=0$. Neglecting resistances as above, we get

$$x^8 - 8x^6 + 20x^4 - 16x^2 + 2 = 0 \tag{S22}$$

with the solutions $x_{1/2}^2 = 2 \pm \sqrt{2+\sqrt{2}}$ and $x_{3/4}^2 = 2 \pm \sqrt{2-\sqrt{2}}$.

Finally, the general case nSE leads to

$$\begin{pmatrix} \lambda_1 & 1 & 0 & & 0 & 0 & 0 \\ 1 & \lambda_2 & 1 & \cdots & 0 & 0 & 0 \\ 0 & 1 & \lambda_2 & & 0 & 0 & 0 \\ & \vdots & & \ddots & & \vdots & \\ 0 & 0 & 0 & & \lambda_2 & 1 & 0 \\ 0 & 0 & 0 & \cdots & 1 & \lambda_2 & 1 \\ 0 & 0 & 0 & & 0 & 1 & \lambda_3 \end{pmatrix} \begin{pmatrix} j_1 \\ j_2 \\ j_3 \\ \vdots \\ j_{n-2} \\ j_{n-1} \\ j_n \end{pmatrix} = i\omega CA \begin{pmatrix} 1 \\ 1 \\ 1 \\ \vdots \\ 1 \\ 1 \\ 1 \end{pmatrix} \tag{S23}$$

Cramer's rule[24] yields the solution

$$j_h = \frac{i\omega CA}{D}\sum_{k=1}^{n} A_{kh}\,, \quad h = 1, 2, \dots, n \tag{S24}$$

with D the determinant of the above matrix and A_{kh} the algebraic complements to the right-hand side unit vector. The resonances occur at the zeros of D which can be found from the secular equation of D. Note that the determinant comprises the main diagonal, the next upper and lower off-diagonals wherein all elements are equal to one, and zero on all remaining positions. As the secular equation of a half-diagonal matrix is given by the product of the main diagonal elements[24], the matrix above is transformed into a half-diagonal obtaining diagonal elements R_k as

$$R_n = \lambda_3, R_{n-1} = \lambda_2 - \frac{1}{R_n}, R_{n-2} = \lambda_2 - \frac{1}{R_{n-1}}, R_{n-3} = \lambda_2 - \frac{1}{R_{n-2}}, \dots,\ R_2 = \lambda_2 - \frac{1}{R_3}, R_1 = \lambda_1 - \frac{1}{R_2} \tag{S25}$$

or

$$R_k = d_k - \frac{1}{R_{k+1}}, \qquad k = 1, 2, \dots, n \tag{S26}$$

with definitions $d_1 = \lambda_1, d_2 = \lambda_2, \dots., d_{n-1} = \lambda_2, d_n = \lambda_3$.

From these recursion formulae, any R_k can be computed, and so can their product which is the secular equation

$$\prod_{k=1}^{n} R_k = 0 \tag{S27}$$

providing resonance frequencies and zero-resistance currents for arbitrary *n*.

From the above equivalent circuit analysis, a symmetry broken *n*SE meta-foil exhibits one main resonance and *n*-1 Fano resonances. At the main resonance, the meta-foil supports the symmetric mode while at the Fano resonances, it supports the antisymmetric modes. If *n* approaches infinity, the main resonance frequency of *n*SE meta-foils tends to the resonance frequency of the S-strings. Besides, from a mechanical point of view, when *n* increases, the mechanical strength of the meta-foil decreases, it becomes more flexible. So, symmetry broken *n*SE meta-foils with *n* > 1 are of interest with regard to both supporting multiple Fano resonances and increasing mechanical flexibility.

Potential for sensing and counterfeiting

Since the very first split ring considerations[25], potential applications of such resonators to sensing were claimed and demonstrated[6,15,26]. Sensing involves two fundamentally different concepts, one detecting a frequency shift in the presence of an agent, the other, the excitation of a resonance by radiation filtered by the agent to be sensed. The *n*SE meta-foils offer advantages as the Fano resonances are much sharper than the main resonance. The smaller peak width is concomitant with lower loss, and can therefore lead to a better resolution of their shift under changes of the dielectric function of a surrounding medium. In terms of sensitivity, the main magnetic peak shift in the meta-foil[13] led already to a value of $5.6 \cdot 10^4$ nm/RIU that is quite close to the maximum value of $5.7 \cdot 10^4$ nm/RIU as claimed[8,10]. Hence, using the Fano peaks of the meta-foil should further improve the sensitivity beyond these values.

Moreover, mechanical vibrations of strings between interconnecting lines can be used to modulate resonance spectra acousto-optically, e.g., by vibration-induced changes of the gap or the area of the capacitors and inductive loops, respectively. Mechanical vibrations may be excited by external mechanical oscillations or by gas or other fluids flowing past the S-strings. Obviously, modulations of resonance frequency by geometric changes that are very slow compared to THz oscillations can be used for sensing deformations and vibrations. Conversely, as the carrier frequency is larger than 1 THz, changes in the dielectric environment or the meta-foil geometry can be detected up to very high frequencies in the GHz range or down to very short times in the sub-ns range. Finally, the well-defined peak frequencies and their ratios in meta-foils with $n \geq 2$ may also serve to identify or code specimen by attaching meta-foils and so protect them from counterfeiting.
